# Associations of changes in neighbourhood walkability with changes in walking activity in older adults: a fixed effects analysis

**DOI:** 10.1186/s12889-021-11368-6

**Published:** 2021-07-06

**Authors:** Erik J. Timmermans, Marjolein Visser, Alfred J. Wagtendonk, J. Mark Noordzij, Jeroen Lakerveld

**Affiliations:** 1 Department of Epidemiology and Data Science, Amsterdam UMC, Vrije Universiteit Amsterdam, Amsterdam Public Health research institute, De Boelelaan 1117, Amsterdam, the Netherlands; 2 Department of Health Sciences, Faculty of Science, Vrije Universiteit Amsterdam, Amsterdam Public Health research institute, Amsterdam, the Netherlands; 3 Upstream Team, www.upstreamteam.nl, Amsterdam UMC, Vrije Universiteit Amsterdam, Amsterdam, the Netherlands; 4 Mulier Institute, Herculesplein 269, Utrecht, the Netherlands

**Keywords:** Built environment, Geographic information systems, Neighbourhood walkability index, Physical activity

## Abstract

**Background:**

Supporting older adults to engage in physically active lifestyles requires supporting environments. Walkable environments may increase walking activity in older adults, but evidence for this subgroup is scarce, and longitudinal studies are lacking. This study therefore examined whether changes in neighbourhood walkability were associated with changes in walking activity in older adults, and whether this association differed by individual-level characteristics and by contextual conditions beyond the built environment.

**Methods:**

Data from 668 participants (57.8–93.4 years at baseline) across three waves (2005/06, 2008/09 and 2011/12) of the Longitudinal Aging Study Amsterdam (LASA) were used. These individuals did not relocate during follow-up. Self-reported outdoor walking activity in minutes per week was assessed using the LASA Physical Activity Questionnaire. Composite exposure measures of neighbourhood walkability (range: 0 (low)-100 (high)) within 500-m Euclidean buffer zones around each participant’s residential address were constructed by combining objectively measured high-resolution Geographic Information System data on population density, retail and service destination density, land use mix, street connectivity, green space density, and sidewalk density. Fixed effects linear regression analyses were applied, adjusted for relevant time-varying confounders.

**Results:**

Changes in neighbourhood walkability were not statistically significantly associated with changes in walking activity in older adults (β_500m_ = − 0.99, 95% CI = -6.17–4.20). The association of changes in neighbourhood walkability with changes in walking activity did not differ by any of the individual-level characteristics (i.e., age, sex, educational level, cognitive impairment, mobility disability, and season) and area-level characteristics (i.e., road traffic noise, air pollution, and socioeconomic status).

**Conclusions:**

This study did not show evidence for an association between changes in neighbourhood walkability and changes in walking activity in older adults. If neighbourhood walkability and walking activity are causally linked, then changes in neighbourhood walkability between 2005/06 and 2011/12 might have been not substantial enough to produce meaningful changes in walking activity in older adults.

## Background

Physical inactivity has been identified as the fourth leading risk factor for global mortality, and increasing physical activity (PA) is crucially important to reduce the risk of developing major chronic non-communicable diseases [1, 2]. Although the health benefits of PA are well-established, the prevalence of insufficient PA among older adults, a rapidly growing population, is alarmingly high. For instance, in the Netherlands, 59.7% of older adults aged 65 years and older did not adherence the PA guidelines in 2019, which points to a considerable scope and need for improvement [3–5]. In particular, walking is an important type of PA in older adults. It is the most preferred activity in older adults, easy to implement in their daily life with low costs and low-risk, and is beneficial to health and functioning [6–8].

It is increasingly recognised that decisions and habits to walk or not are partly driven by contextual determinants; for instance, through factors in the built environment that hinder or enable walking [6, 9]. Older adults may be particularly susceptible to environmental factors in the residential environment as they are likely to spend more time closer to home than younger adults [10]. Identifying modifiable characteristics of the built environment associated with walking in older adults is important to inform interventions and policy measures supporting healthy and active ageing [6]. To date, research on the associations of the neighbourhood built environment with walking activity in older adults is scarce, not generalizable to Western European countries, and limited by methodological and conceptual issues [6, 11–13].

Literature reviews have shown that most studies have examined associations of single built environmental characteristics with older adults’ walking activity and do not take into account that individuals are exposed to multiple environmental characteristics at the same time, that these characteristics are likely to interact with each other, and individually might have relatively little influence [6, 11, 12]. It has therefore been argued that environmental exposures should be studied in combination rather than with a traditional single exposure approach [14]. Neighbourhood walkability reflects the degree to which neighbourhoods are conducive to walking activity. It is measured by an index combining several key spatial components that particularly facilitate walking, such as population density, retail and service destination density, land use mix, street connectivity, green space density, and sidewalk density [6, 15–22]. Previous studies examining the association between neighbourhood walkability and older adults’ PA, including walking, generally show a positive association [6, 11, 12]. These studies have predominantly been conducted in Australia and the United States of America (USA), and the results may not be applicable to Western European countries as large differences in built environmental design exist between countries [6, 11–13].

Several studies investigating associations of neighbourhood walkability with PA measures in older adults rely on traditional neighbourhood data that are the result of data aggregation on an existing (administrative) spatial scale, such as census areas [6, 12]. However, such areas are often based on arbitrarily defined boundaries used to aggregate continuous spatial features. Several problems associated with such area-level data have been described in detail in the environmental-health literature, including the Modifiable Areal Unit Problem [23]. The essence of the Modifiable Areal Unit Problem is that analytical results for the same data in the same area can be different, if aggregated in different ways [23]. The present study tries to limit this methodological problem by considering the individual’s actual residential location and using objectively measured high-resolution exposure data to define walkability in Euclidean buffer zones around each individual’s residential address [23, 24].

Moreover, existing studies assessing associations of neighbourhood walkability with PA measures in older adults are based on cross-sectional data and cannot assess a temporal relationship between exposure and outcome [6, 11, 12]. This raises the question whether an increase in neighbourhood walkability over time is associated with increased walking activity among older adults, and longitudinal studies are required to address this question [6, 11, 12, 25]. Two major concerns in cross-sectional as well as longitudinal environment-PA studies are bias by residential self-selection and unmeasured confounding [6, 11, 12, 25]. Residential self-selection is the phenomenon that individuals choose where to live based on their needs and preferences [25]. Consequently, associations between walkable neighbourhoods and walking activity may be more pronounced because individuals who like to walk choose to live in more walkable areas [25]. The present study effectively addresses these concerns by analysing longitudinal data of non-relocators in fixed effects models that estimate effects based on within-person comparisons over time and eliminate bias due to confounding by measured and unmeasured time-invariant characteristics, including personal preferences; assuming that these remain stable over time [25–28].

To increase the effectiveness of future area-based PA promotion approaches, studies are needed that assess the neighbourhood walkability-PA association in different subgroups of older adults and in different objectively measured social and physical contextual (i.e., area-level) conditions [6, 11, 12]. Socio-ecological models suggest that changes in environmental characteristics are more strongly related to changes in PA in socially and physically vulnerable subgroups, e.g., defined by women, older age, lower socioeconomic position, cognitive impaired and lower functional ability [9, 29–34]. Furthermore, these models suggest that the impact of built environmental characteristics on PA could be strengthened or attenuated by contextual conditions beyond the built environment, such as road traffic noise, air pollution, and socioeconomic status [9].

This longitudinal study extends previous research by examining whether changes in neighbourhood walkability are associated with changes in walking activity in older adults in the Netherlands, and whether this association differs by individual-level characteristics and contextual conditions. It is hypothesised that increased neighbourhood walkability levels are associated with increased walking activity over time in older adults. It is expected that this association is stronger in socially and physically more vulnerable subgroups. Furthermore, it is expected that better contextual conditions, such as lower levels of road traffic noise and air pollution, and higher levels of area-level socioeconomic status, strengthen the positive association of neighbourhood walkability with walking activity in older adults.

## Methods

### Design and study sample

Data from the Longitudinal Aging Study Amsterdam (LASA) were used in this study. LASA is an ongoing, prospective cohort study in the Netherlands on the determinants, trajectories and consequences of physical, cognitive, emotional and social functioning in older adults. Details on the LASA sampling and measurements have been described previously [35, 36]. In short, a random sample of older men and women (55–85 years), stratified for age and sex, was drawn from the population registries of 11 municipalities in three geographic regions that together represent the socio-cultural variety in the Netherlands: the Protestant North-East, the Roman Catholic South, and the secularised West. The baseline data collection was conducted in 1992/93 and the baseline sample included 3107 respondents. Since then, follow-up measurements have been conducted approximately every 3 years. An additional sample of 1002 respondents aged 55 to 64 years was drawn in 2002/03 using the same sampling frame as the original cohort.

For this study, data from waves 2005/06, 2008/09 and 2011/12 were used. The sample in 2005/06 included 2165 respondents, and 643 of which were lost to follow-up by 2011/12 (n_deceased_ = 449, n_refusal_ = 129, n_ineligible_ = 57, n_not_contacted_ = 8) [37]. Respondents with no data on their residential address during the study period (*n* = 3) were excluded from the analyses. Furthermore, respondents who relocated during follow-up (*n* = 314) were additionally excluded from the main analyses, as moving is often a result of changes in individual-level circumstances that likely confound the neighbourhood walkability-walking association [25]. In addition, respondents with missing data on walking activity (*n* = 489), the neighbourhood walkability exposure measure (*n* = 15), or on at least one confounder or effect modifier (*n* = 23) were excluded from the analyses. Respondents with extreme outliers on the walking activity outcome measure (i.e., ≥3 Standard Deviations (SDs) from the mean; *n* = 10) were also excluded from the analyses. The final analytical sample consisted of 668 individuals with full data at all three waves. At the LASA study wave that acted as baseline for the current study (i.e., wave 2005/06), the included participants lived in 34 municipalities across the Netherlands, which consisted of 467 municipalities in 2005.

At baseline, the included participants were younger and higher educated than the excluded individuals. The proportion of individuals with a cognitive impairment and mobility disability was lower in the included group than the excluded group. The included participants completed the LASA Physical Activity Questionnaire (LAPAQ) more often in autumn and winter, while the excluded individuals completed this questionnaire more often in summer. The included group did not differ from the excluded group in terms of neighbourhood walkability (components), walking activity, exposure to road traffic noise and air pollution, and area-level socioeconomic status.

All participants completed an informed consent. LASA was approved by the Ethical Review Board of the VU University medical center and is conformed to the principles embodied in the Declaration of Helsinki.

#### Dependent variable

##### Walking activity

Walking activity was defined as the time spent on walking outside in minutes per week, and was measured using the LAPAQ [38]. The LAPAQ captures how often participants participated in various activities, including walking outside, in the previous 2 weeks (i.e., frequency) and for how long they usually did this each time (i.e., duration). In order to calculate the time spent on walking outside in minutes per week, the frequency and duration of walking outside were multiplied and divided by two. The LAPAQ is considered to be a valid and reliable instrument for classifying PA in older adults [38].

#### Independent variables

##### Neighbourhood walkability index and components

In the Geoscience and Health Cohort Consortium (GECCO), a neighbourhood walkability index has been constructed for various exposure areas covering the whole of the Netherlands [39, 40]. The neighbourhood walkability index is a composite measure originally combining three spatial components, including population density, land use mix, and street connectivity [16]. Recently, it has been argued that other components, such as retail and service destination density, green space density, and sidewalk density, are also relevant built environmental attributes for walking and should be included in the neighbourhood walkability index [22]. The construction of the neighbourhood walkability index in GECCO has been described in detail elsewhere, and this index is based on the following six spatial components: (1) population density, (2) retail and service destination density, (3) land use mix, (4) street connectivity, (5) green space density, and (6) sidewalk density [22, 39].

The relevance of these six components for walkability is evidence-informed and the various components are based on the most detailed geo-data available with national coverage over a substantial time period [22]. Population density was defined as the number of residents per hectare, based on data from Statistics Netherlands [41]. Retail and service destination density was defined as the percentage of area devoted to retail, hospitality and catering industry, and social services (e.g., schools, medical services, religious buildings), based on land use data from Statistics Netherlands [42]. Land use mix was assessed using the entropy score − 1^*^Σ_k_(p_k_∗ln(p_k_))/ln(N), where p is the proportion of area devoted to a specific land use category (i.e., k), and N is the number of (aggregated or grouped) land use categories included [43]. Data on the following land use categories were obtained from Statistics Netherlands: (1) residential areas, (2) commercial areas, (3) social-cultural services, (4) offices and public services, and (5) green space and recreation [42]. Street connectivity was defined as the number of intersections (i.e., three or more legs) of roads accessible for pedestrians per hectare. The data on street connectivity were retrieved from the topographical (TOP10) map in the Basic Topography Register System of The Netherlands’ Cadastre, Land Registry and Mapping Agency [44]. Green space density was defined as the percentage of area devoted to green space (i.e., parks, public gardens, forests, and cemeteries). The data on green space were retrieved from Statistics Netherlands [42]. Sidewalk density was defined as the percentage of area devoted to sidewalks, and the relevant data were derived from the Key Register Large-scale Topography of the Netherlands Ministry of Infrastructure and Environment [22, 45].

All six components were computed as raster cell values in a regular spaced grid covering the Netherlands with raster cells of 25 × 25 m. The individual raster cell values for each component were calculated using focal statistics, which means that the value of each output cell is a function of the values of all input cells that are in a specified Euclidean buffer around that cell. For each component, all raster cell values were standardised (i.e., converted into z-scores). Subsequently, the (un)standardised raster cell values were extracted and linked to the X- and Y-coordinates of all addresses in the Netherlands, using Geo Data and Model Software (GeoDMS) (Object Vision BV, Amsterdam, the Netherlands). To create the neighbourhood walkability index for each address, the linked standardised values were summed. Finally, the sum score was rescaled such that the neighbourhood walkability index ranged between 0 and 100, with higher scores representing higher neighbourhood walkability levels [22]. No weights were applied to the components of the neighbourhood walkability index, since an equally weighted index seems to perform well in a Dutch context [46, 47].

In the present study, we derived the neighbourhood walkability index, and its (un)standardised components, from 500-m Euclidean buffer zones. This buffer zone size is often used in environment-PA research and is considered to be a relevant spatial context for older adults’ walking [6]. In the present study, we linked the constructed neighbourhood walkability indices for the years 2005, 2008 and 2011, and their related (un)standardised components, to the residential address of participants at waves 2005/06, 2008/09 and 2011/12, respectively. Due to data-availability limitations, some components were related to a nearby year. More specifically, the neighbourhood walkability indices in 2005, 2008 and 2011 were based on population density (in 2005, 2008 and 2011), retail and service destination density (in 2006, 2008 and 2010), land use mix (in 2006, 2008 and 2010), street connectivity (in 2003 (for 2005), 2003 (for 2008) and 2012), green space density (in 2006, 2008 and 2010), and sidewalk density (in 2003, 2008 and 2012) [22].

#### Potential individual-level effect modifiers

The following potential individual-level effect modifiers were considered: age, sex, educational level, cognitive impairment, mobility disability, and season.

Information on *age* in years and *sex* (0 = man, 1 = woman) was derived from population registries. For the analyses that assess effect modification, age was dichotomised based on the median per wave (0 = younger-old (≤median), 1 = older-old (>median)).

*Educational level* was dummy-coded (0 = no, 1 = yes) into: low (reference category; elementary education not completed, elementary education, or lower vocational education), intermediate (general intermediate education, intermediate vocational education, or secondary education), and high (higher vocational education, college education, or university education).

General cognitive functioning was measured using the Mini-Mental State Examination (MMSE) [48, 49]. The MMSE is a 23-item global cognitive function test, which includes questions on orientation in time and place, attention, language, memory and visual construction. The total MMSE score ranges from 0 to 30, with a higher score indicating better performance. *Cognitive impairment* was considered as present (0 = no, 1 = yes) when participants’ MMSE score was below 23 [49].

*Mobility disability* was assessed as the self-reported degree of difficulty or need of help with walking five minutes outdoors without resting. Mobility disability was considered as present (0 = no, 1 = yes) when participants indicated that they could not do this activity at all or without help [50].

The astronomical *season* in which the LAPAQ was completed by participants was dummy-coded (0 = no, 1 = yes) into: winter (reference category), spring, summer, and autumn.

#### Potential area-level effect modifiers

The following potential area-level effect modifiers were considered: road traffic noise, air pollution, and socioeconomic status.

Data on daily average *road traffic noise* exposure were retrieved from the PBL Netherlands Environmental Assessment Agency [51]. These data were modelled for various years in raster cells of 25 × 25 m covering the Netherlands, and operationalised as Level day-evening-night (Lden), expressed in A-weighted decibels (dB(A)) [51, 52]. In GECCO, the raster cell values have been extracted and linked to the X- and Y-coordinates of all addresses in the Netherlands, using GeoDMS (Object Vision BV, Amsterdam, the Netherlands) [40]. In this study, we linked road traffic noise data in 2004, 2008 and 2011 to the residential address of participants at waves 2005/06, 2008/09 and 2011/12, respectively.

Data on annual average outdoor *air pollution* concentrations at the participants’ residential address were estimated by land use regression models for the year 2009 by the Institute for Risk Assessment Sciences as part of the European Study of Cohorts for Air Pollution Effects, as described elsewhere [53–55]. In this study, we used data of the annual average concentrations of the mean blackness of PM2.5 filters, which is a proxy for elemental carbon (i.e., soot), the dominant light absorbing substance. Previous studies confirm the stability of spatial contrasts in air pollution levels over periods of 7 years and longer [56–58].

Area-level *socioeconomic status* scores of four-digit postal code areas were retrieved from the Netherlands Institute for Social Research [59]. In the Netherlands, four-digit postal code areas (average area size: 8.3 km^2^) are geographically delineated administrative areas and include, on average, approximately 1870 households [40]. The socioeconomic status scores are based on the average income, the percentage of residents with a low income, the percentage of residents with a low level of education, and the percentage of unemployed residents in these areas [59, 60]. Higher scores indicate a higher area-level socioeconomic status. The socioeconomic status scores in 2006 were linked to participants’ data at waves 2005/06 and 2008/09, and the socioeconomic status scores in 2010 were linked to participants’ data at wave 2011/12.

For the analyses that assess effect modification, the levels of road traffic noise, air pollution, and socioeconomic status, were dichotomised based on the wave-specific median (0 = lower level (≤median), 1 = higher level (>median)).

#### Potential confounders

The cross-sectional analyses of baseline data were adjusted for *educational level*, *cognitive impairment*, *mobility disability*, *season*, area-level *road traffic noise*, *air pollution*, and *socioeconomic status*.

For the fixed effects analyses, the confounder *time* was constructed, indicating wave number (wave 2005/06 = 1, wave 2008/09 = 2, and wave 2011/12 = 3) [24]. *Cognitive impairment*, *mobility disability* and *season* (if no effect modifiers) were included as individual-level time-varying confounders in the fixed effects analyses. These confounders were measured at all three waves, capturing changes that occurred during follow-up.

*Age* and *sex* were considered to be no confounders in the association between neighbourhood walkability and walking activity. *Educational level* was considered to be time-invariant because of the relatively old age of our study sample, and was therefore not included in the fixed effects analyses [61]. Area-level *road traffic noise*, *air pollution*, and *socioeconomic status* are relatively stable over time in the Netherlands, and were also considered to be time-invariant in this study [56–58, 62, 63].

### Statistical analyses

Characteristics of the study sample and (within-person changes between waves in) the area-level exposure measures are presented using descriptive statistics. Means and SDs are presented for normally distributed continuous variables. Median and interquartile range (IQR) are presented for skewed distributed continuous variables. Frequencies and percentages are presented for dichotomous or categorical variables. Pearson correlations were assessed between the neighbourhood walkability index and all standardised neighbourhood walkability components at baseline. As a first step, cross-sectional associations of neighbourhood walkability, and its (un)standardised components, with walking activity at baseline, were tested using linear regression analyses, adjusting for educational level, cognitive impairment, mobility disability, season, area-level road traffic noise, air pollution, and socioeconomic status.

To study possible effect modification by age, sex, educational level, cognitive impairment, mobility disability, season and area-level road traffic noise, air pollution, and socioeconomic status, an interaction term between the neighbourhood walkability index and each potential effect modifier was created. Each interaction term, together with its two main terms and time, were included in a fixed effects model. Because the power of statistical tests for higher order terms is generally lower than for first-order terms, the interaction term in this model was considered to be statistically significant at a *p*-value below 0.10 [64, 65]. In case of a statistically significant interaction term, stratified analyses were conducted.

Fixed effects analyses were used to examine the association between within-person change in neighbourhood walkability and within-person change in walking activity. A fixed effects analysis adjusts for potential confounders that do not change over time, but vary between individuals. Provided that changes are observed, a fixed effects analysis is able to capture to what extent changes in neighbourhood walkability between time-points are related to changes in walking activity between time-points [26–28]. In this study, two fixed effects models were applied: a linear-regression model adjusting for time only (i.e., the crude model), and a model with additional adjustment for time-varying characteristics (i.e., cognitive impairment, mobility disability and season) (if no effect modifiers). Fixed effects analyses were also conducted to assess the associations of changes in the (un)standardised components of the neighbourhood walkability index with changes in walking activity, separately. In all fixed effects analyses, robust standard errors were used to account for non-independence clustering at the individual-level. The following model was used for the analyses:

$$ {\mathrm{Walking}}_{it}={\upmu}_t+{\upbeta}_1{\mathrm{neighbourhood}\ \mathrm{walkability}}_{it}+{\upbeta}_2{\mathrm{x}}_{it}+{\upalpha}_i+{\upepsilon}_{it} $$

where Walking_*it*_ indicates walking activity for individual *i* at time *t*, μ_t_ accounts for time effects that are fixed for all individuals, neighbourhood walkability_*it*_ represents the neighbourhood walkability exposure measure (i.e., the neighbourhood walkability index or its (un)standardised components), x_*it*_ is a vector of time-varying control regressors, α_*i*_ controls for time-invariant characteristics, while ϵ_*it*_ is the error term.

In order to examine the robustness of our findings, the association of changes in neighbourhood walkability with changes in walking activity was assessed in sensitivity analyses in which the neighbourhood walkability index was derived from smaller (i.e., 250-m) and larger (i.e., 1000-m and 2000-m) Euclidian buffer zones.

In all analyses, except in the analyses testing the interaction terms, a *p*-value below 0.05 was considered as statistically significant. The descriptive analyses were performed in IBM SPSS Statistics (Version 26; IBM Corp, Armonk, New York, USA). The fixed effects analyses were performed using STATA (Version 14; StataCorp LP, College Station, Texas, USA).

## Results

The baseline characteristics of the study sample and area-level exposure measures are presented in Table 1. The mean age was 67.7 (SD = 7.2) years with an age-range of 57.8–93.4 years, and 53.4% were women. The mean follow-up time was 6.1 (SD = 0.2) years, ranging from 5.2 to 6.8 years. On average, the participants spent 206.0 (SD = 233.6) minutes on walking outside per week. The average neighbourhood walkability index was 30.1 (SD = 15.5), ranging from 0.0 to 71.5. In general, moderate to strong positive correlations were observed between the neighbourhood walkability index and all standardised neighbourhood walkability components at baseline (Table 2).

**Table 1:** Description of the study sample and area-level exposure measures at baseline (wave 2005/06)

Variables	Study sample (***n*** = 668)	
	Mean ± SD ^**a**^	Range
**Walking activity**		
Walking outside in minutes/week	206.0 ± 233.6	4.0–2100.0
**Neighbourhood walkability** ^**b**^		
Neighbourhood walkability index	30.1 ± 15.5	0.0–71.5
**Unstandardised neighbourhood walkability components** ^**b**^		
Population density	47.29 ± 38.10	0.19–230.43
Retail and service destination density	3.46 ± 5.67	0.00–34.70
Land use mix	0.36 ± 0.17	0.00–0.87
Street connectivity	1.17 ± 0.52	0.00–2.70
Green space density	8.31 ± 8.06	0.00–58.89
Sidewalk density	6.77 ± 4.59	0.00–18.90
**Standardised neighbourhood walkability components** ^**b**^		
Population density	5.08 ± 4.28	−0.20-25.64
Retail and service destination density	1.17 ± 2.20	−0.18-13.27
Land use mix	2.46 ± 1.32	− 0.34-6.38
Street connectivity	4.14 ± 2.02	− 0.40-10.07
Green space density	0.04 ± 0.45	−0.42-2.89
Sidewalk density	4.68 ± 3.33	− 0.24-13.47
**Time-invariant characteristics**		
Sex (%)		
Men	46.6	
Women	53.4	
Educational level (%)		–
Low	41.0	
Intermediate	34.1	
High	24.9	
**Time-varying characteristics**		
Age in years	67.7 ± 7.2	57.8–93.4
Cognitive impairment (%)		–
No	97.9	
Yes	2.1	
Mobility disability (%)		–
No	94.2	
Yes	5.8	
Season (%)		–
Winter	26.9	
Spring	27.7	
Summer	13.2	
Autumn	32.2	
**Area-level exposure measures**		
Road traffic noise in dB(A) (Median (IQR))	52.0 (48.2–56.5)	–
Air pollution: PM2.5 absorbance in 10^−5^ m^−1^ (Median (IQR))	1.19 (1.04–1.36)	–
Socioeconomic status score (Median (IQR))	0.32 (− 0.22–0.50)	–

^a^ Mean ± SD are presented, unless stated otherwise

^b^ The neighbourhood walkability index (index range: 0–100) and the (un)standardised components were derived from 500-m Euclidean buffer zones around the residential address of participants. The units of measures of the unstandardised components are: (1) population density [number of residents/hectare], (2) retail and service destination density [percentage of area devoted to retail and services], (3) land use mix [entropy index range: 0–1], (4) street connectivity [number of intersections (three or more legs) of roads accessible by pedestrians/hectare], (5) green space density [percentage of area devoted to green space], and (6) sidewalk density [percentage of area devoted to sidewalks]. The standardised components are all z-scores

**Table 2:** Pearson correlations between the neighbourhood walkability index and all standardised neighbourhood walkability components at baseline (wave 2005/06) ^a^

Neighbourhood walkability (components) ^**b**^	1	2	3	4	5	6	7
1. Neighbourhood walkability index	1.00						
2. Population density	**.85**	1.00					
3. Retail and service destination density	**.54**	**.15**	1.00				
4. Land use mix	**.42**	.01	**.59**	1.00			
5. Street connectivity	**.71**	**.54**	**.22**	**.22**	1.00		
6. Green space density	**.12**	−.07	.03	**.38**	**.14**	1.00	
7. Sidewalk density	**.94**	**.84**	**.39**	**.28**	**.59**	.05	1.00

^a^ In bold: p-value< 0.01

^b^ The neighbourhood walkability index (index range: 0–100) and the standardised components (z-scores) were derived from 500-m Euclidean buffer zones around the residential address of participants

### Cross-sectional analyses

The cross-sectional analyses showed that neighbourhood walkability (β_500m_ = 0.76, 95% Confidence Interval (CI) = − 0.86–2.37), and its (un)standardised components (results not shown), were not statistically significantly associated with walking activity in older adults at baseline.

### Within-person changes

Within-person changes between two subsequent waves were observed for the outcome measure and all exposure measures, consisting of both increases and decreases in measures over time (Table 3). For walking in minutes/week, 92.9% of the available 1336 person observations exhibited changes. There was an average negative change in walking activity, i.e., a decrease of 2.6 min per week (SD = 287.2; range: − 2065.0-2047.5). Of all neighbourhoods where participants resided, 93.9% exhibited changes in walkability within the 500-m Euclidean buffer zones. The average change in neighbourhood walkability was, however, nihil, i.e., an increase of 0.3 (SD = 2.1; range: − 13.0-19.3). The average changes in the separate (un)standardised neighbourhood walkability components were also small. For instance, the largest average change that was observed in the standardised neighbourhood walkability components was 0.15 (SD = 0.84; range: − 6.84-11.50), and was related to retail and service destination density.

**Table 3:** Within-person changes in walking activity, neighbourhood walkability and its (un)standardised components between each of the three waves (2005/06, 2008/09 and 2011/12)

	Decrease			No change			Increase			Average change	
	n	Mean ± SD	Range	n	Mean ± SD	Range	n	Mean ± SD	Range	n	Mean ± SD
**Walking activity**											
Walking outside in minutes/week	625	− 175.8 ± 240.7	−2065.0- -2.5	95	0.0 ± 0.0	0.0–0.0	616	172.8 ± 242.8	1.0–2047.5	1336	−2.6 ± 287.2
**Neighbourhood walkability** ^**a**^											
Neighbourhood walkability index	707	− 0.7 ± 0.9	−13.0 - -0.1	82	0.0 ± 0.0	0.0–0.0	547	1.8 ± 2.4	0.1–19.3	1336	0.3 ± 2.1
**Unstandardised neighbourhood walkability components** ^**a**^											
Population density	741	−1.00 ± 1.71	−21.07 - -0.01	16	0.00 ± 0.00	0.00–0.00	579	1.75 ± 2.89	0.01–33.80	1336	0.21 ± 2.66
Retail and service destination density	102	−1.33 ± 2.17	−17.43- -0.07	862	0.00 ± 0.00	0.00–0.00	372	3.01 ± 3.89	0.07–36.52	1336	0.74 ± 2.58
Land use mix	200	−0.03 ± 0.04	− 0.33- -0.01	703	0.00 ± 0.00	0.00–0.00	433	0.07 ± 0.07	0.01–0.41	1336	0.02 ± 0.06
Street connectivity	8	−0.06 ± 0.07	−0.22- -0.01	787	0.00 ± 0.00	0.00–0.00	541	0.09 ± 0.09	0.01–0.78	1336	0.04 ± 0.07
Green space density	299	−1.12 ± 2.20	−23.87- − 0.07	813	0.00 ± 0.00	0.00–0.00	244	1.07 ± 1.53	0.07–9.24	1336	-0.07 ± 1.39
Sidewalk density	172	−0.22 ± 0.32	− 2.42- -0.01	382	0.00 ± 0.00	0.00–0.00	782	0.18 ± 0.32	0.01–5.84	1336	0.08 ± 0.30
**Standardised neighbourhood walkability components** ^**a**^											
Population density	807	−0.13 ± 0.19	−2.35- − 0.01	72	0.00 ± 0.00	0.00–0.00	457	0.19 ± 0.33	0.01–3.70	1336	-0.01 ± 0.29
Retail and service destination density	460	− 0.26 ± 0.48	−6.84- -0.01	304	0.00 ± 0.00	0.00–0.00	572	0.56 ± 1.08	0.01–11.50	1336	0.15 ± 0.84
Land use mix	725	− 0.13 ± 0.21	−2.60- -0.01	231	0.00 ± 0.00	0.00–0.00	380	0.50 ± 0.49	0.01–2.99	1336	0.07 ± 0.41
Street connectivity	542	− 0.32 ± 0.19	−1.33- -0.01	672	0.00 ± 0.00	0.00–0.00	122	0.32 ± 0.37	0.01–2.20	1336	−0.10 ± 0.26
Green space density	783	−0.08 ± 0.09	− 1.42- -0.01	483	0.00 ± 0.00	0.00–0.00	70	0.08 ± 0.09	0.01–0.39	1336	−0.04 ± 0.09
Sidewalk density	958	−0.12 ± 0.14	−1.91- -0.01	97	0.00 ± 0.00	0.00–0.00	281	0.18 ± 0.32	0.01–4.08	1336	−0.05 ± 0.22

^a^ The neighbourhood walkability index (index range: 0–100) and the (un)standardised components were derived from 500-m Euclidean buffer zones around the residential address of participants. The units of measures of the unstandardised components are: (1) population density [number of residents/hectare], (2) retail and service destination density [percentage of area devoted to retail and services], (3) land use mix [entropy index range: 0–1], (4) street connectivity [number of intersections (three or more legs) of roads accessible by pedestrians/hectare], (5) green space density [percentage of area devoted to green space], and (6) sidewalk density [percentage of area devoted to sidewalks]. The standardised components are all z-scores

### Fixed effects analyses

Fixed effects analyses showed statistically non-significant negative associations between changes in neighbourhood walkability and changes in walking activity (Crude model: β_500m_ = − 0.93, 95% CI = -6.15–4.29; Adjusted model: β_500m_ = − 0.99, 95% CI = -6.17–4.20) (Table 4). The result from the adjusted model suggests that a 10-unit increase in the neighbourhood walkability index is associated with a decrease in walking activity of 9.90 min, albeit statistically non-significant. There was no statistical evidence that the association of changes in neighbourhood walkability with changes in walking activity differed by the individual-level characteristics age, sex, educational level, cognitive impairment, mobility disability, and season nor by the area-level characteristics road traffic noise, air pollution, and socioeconomic status (i.e., the *p*-values of all relevant interaction terms were ≥ 0.10).

**Table 4:** Fixed effects linear regression models regressing changes in walking in minutes per week on changes in neighbourhood walkability, and its (un)standardised components, using data from waves 2005/06, 2008/09 and 2011/12

	Crude model ^**a**^	Adjusted model ^**b**^
	**Beta (95% CI)**	**Beta (95% CI)**
**Neighbourhood walkability** ^**c**^		
Neighbourhood walkability index	−0.93 (− 6.15–4.29)	−0.99 (− 6.17–4.20)
**Unstandardised neighbourhood walkability components** ^**c**^		
Population density	−1.64 (− 5.31–2.03)	−1.25 (− 4.99–2.49)
Retail and service destination density	− 0.57 (− 3.87–2.73)	− 0.64 (− 3.89–2.61)
Land use mix	38.53 (− 194.92–271.98)	31.05 (− 201.83–263.93)
Street connectivity	43.18 (− 155.63–241.99)	45.78 (− 151.79–243.35)
Green space density	4.04 (− 5.52–13.61)	3.54 (− 6.25–13.33)
Sidewalk density	− 2.71 (− 41.40–35.98)	−5.15 (− 43.05–32.75)
**Standardised neighbourhood walkability components** ^**c**^		
Population density	−17.73 (−48.83–13.37)	−14.54 (− 46.16–17.07)
Retail and service destination density	−2.31 (− 12.65–8.04)	−2.37 (− 12.67–7.93)
Land use mix	5.59 (− 25.27–36.46)	4.40 (− 26.47–35.28)
Street connectivity	− 11.75 (− 68.08–44.59)	− 11.74 (− 68.26–44.77)
Green space density	48.79 (− 106.17–203.75)	40.80 (− 117.44–199.03)
Sidewalk density	− 8.43 (− 57.26–40.40)	− 12.08 (− 60.38–36.23)

^a^ The crude model is a fixed effects linear regression model adjusting for time only

^b^ The adjusted model is a fixed effects linear regression model adjusting for time, cognitive impairment, mobility disability, and season

^c^ The neighbourhood walkability index (index range: 0–100) and the (un)standardised components were derived from 500-m Euclidean buffer zones around the residential address of participants. The units of measures of the unstandardised components are: (1) population density [number of residents/hectare], (2) retail and service destination density [percentage of area devoted to retail and services], (3) land use mix [entropy index range: 0–1], (4) street connectivity [number of intersections (three or more legs) of roads accessible by pedestrians/hectare], (5) green space density [percentage of area devoted to green space], and (6) sidewalk density [percentage of area devoted to sidewalks]. The standardised components are all z-scores

Fixed effects analyses also indicated that changes in (un)standardised neighbourhood walkability components were not statistically significantly associated with changes in walking activity in older adults (Table 4).

### Sensitivity analyses

The sensitivity analyses including neighbourhood walkability indices that were derived from 250-, 1000-, and 2000-m Euclidean buffer zones did not reveal substantially different results compared to the main analyses in which the included neighbourhood walkability index was derived from a 500-m Euclidean buffer zone (Crude models: β_250m_ = 0.16, 95% CI = -3.51–3.83; β_1000m_ = − 1.98, 95% CI = -7.40–3.45; β_2000m_ = − 1.45, 95% CI = -10.41–7.50; Adjusted models: β_250m_ = 0.44, 95% CI = -3.19–4.06; β_1000m_ = − 2.04, 95% CI = -7.40–3.31; β_2000m_ = − 1.94, 95% CI = -10.77–6.88).

## Discussion

This study examined whether changes in neighbourhood walkability over time were associated with changes in time spent on walking per week in older adults, and whether this association differed by individual-level characteristics and contextual conditions. By examining whether actual changes in the neighbourhood built environment are associated with changes in walking activity in older adults, the present study addresses a highly relevant question for policymakers who aim to increase healthy and active ageing in society [6]. In contrast with our expectations, we found statistically non-significant negative associations between changes in neighbourhood walkability and changes in walking activity in older adults. Furthermore, we found that the association of changes in neighbourhood walkability with changes in walking activity in older adults did not differ by any of the individual-level characteristics (i.e., age, sex, educational level, cognitive impairment, mobility disability, and season) and area-level characteristics (i.e., road traffic noise, air pollution, and socioeconomic status).

### Strengths and limitations

To our knowledge, this is the first longitudinal study that examines the association of changes in neighbourhood walkability with changes in walking activity in older adults in the Netherlands, and whether this association differed by individual-level characteristics and contextual conditions [6, 11, 12]. An innovative aspect of the present study is the use of a composite exposure measure of neighbourhood walkability that combines objectively measured high-resolution Geographic Information System data on six components of the built residential environment facilitating walking [14, 22]. An important strength of the present study is the use of individual-level longitudinal walkability data in fixed effects analyses, limiting the effect of geographical-methodological issues related to linking area-level exposure measures to individual-level outcome measures, and reducing spatial misclassification [23, 24, 28, 66]. Furthermore, the fixed effects approach eliminates the effects of unmeasured time-invariant characteristics and therefore alleviates omitted-variable bias [24–28].

This study has several limitations to consider. Firstly, we were not able to integrate all components of the built environment that are relevant for older adults’ walking into the neighbourhood walkability index. For instance, the index does not integrate data on pedestrian crossing availability, presence of street furniture, and type and quality of footpath pavement, because these are currently not available for the entire country and/or for the required time period [6, 22, 67]. For the same reasons, the index does not integrate data on relevant components of the social environment, such as crime-related safety [6, 22, 68]. Secondly, the outcome measure on walking activity was self-reported. Although this measure was based on a validated questionnaire, it might have caused recall bias as it is difficult for older adults to provide accurate measures of their walking activity [38, 69]. Thirdly, the fixed effects approach does not remove the potential bias of time-varying confounders [26, 27]. We addressed this limitation by adjusting our fixed effects analyses for relevant measured time-varying confounders, but we were not able to control for all potentially relevant factors (e.g., household income was not included in the present analyses due to a substantial amount of missing data). Finally, as fixed effects models only rely on within-person changes, they ignore between-individual effects and have less statistical power [24, 26, 27]. In addition, fixed effects analyses require multiple measurements. This may have resulted in the inclusion of a relatively healthy group of older adults at baseline, which may limit the generalizability of our findings.

### Potential explanations for the study findings

Area-level factors that were not considered in this study, such as safety and social cohesion, may have affected whether or not older adults walk in their residential area, and may have concealed the associations between changes in neighbourhood walkability and changes in walking activity. Furthermore, perceptions of neighbourhood walkability (and its components) might be more important for older adults’ walking activity than objectively measured area-level walkability (and its components), which individuals may not be aware of [28, 70–72]. In line with other environmental-health studies using fixed effects analyses, the present study shows that the built environment only changes marginally over a number of years [24, 25, 28]. An explanation for our null findings is thus that a follow-up time of six years might have been too short. The changes in neighbourhood walkability, and its components, between 2005/06 and 2011/12 might have been not substantial enough to produce meaningful changes in walking activity in older adults.

Based on socio-ecological models, we expected that changes in neighbourhood walkability were more strongly associated with changes in walking activity in socially and physically more vulnerable subgroups, and that this association would also be modified by contextual conditions beyond the built environment [9, 29–34]. A more walkable residential area includes fewer environmental barriers and more opportunities for walking, and may invite socially and physically more vulnerable older adults more strongly to participate in walking activities, while less vulnerable older adults might be less susceptible to such changes [21, 30, 31]. Furthermore, a more walkable residential area may invite older adults more strongly to participate in walking activities if other non-built contextual factors also facilitate/encourage walking [9]. However, the present study did not show statistical evidence that the association of changes in neighbourhood walkability with changes in walking activity differed by any of the included individual-level and area-level effect modifiers. These findings need replication in future research, including larger numbers of participants per subgroup and contextual condition.

### Suggestions for future research

To obtain more insight into the association between changes in neighbourhood walkability and PA in older adults, future studies could replicate our approach with longitudinal data from a larger number of participants over a longer follow-up time, with more substantial environmental changes over time. Furthermore, (quasi-)natural experiments evaluating the impact of urban regeneration programs, focusing on improving aspects of the built environment in residential areas, on neighbourhood walkability and walking behaviours of older adults may also be informative to the causal relationship between neighbourhood walkability and walking in this group [73]. In addition, future studies could consider to incorporate other factors from the objective built as well as social environment relevant to walking in the neighbourhood walkability index, and to assess the role of (changes in) perceived environmental characteristics. Compared to Euclidean buffer zones, network buffer zones, that define areas accessible via a street network within a specified distance, might better capture network constrained attributes, such as retail and service destination density and sidewalk density [61, 74]. Future efforts could be made to incorporate such network-based data in the neighbourhood walkability index.

## Conclusions

In contrast with previous cross-sectional studies, our baseline cross-sectional analysis did not provide statistical evidence for an association of neighbourhood walkability with walking activity in older adults [6, 11, 12]. Moreover, the findings of our fixed effects analyses did also not show statistical evidence for an association between changes in neighbourhood walkability and changes in walking activity in older adults. If neighbourhood walkability and walking activity are causally linked, then observed changes in neighbourhood walkability between 2005/06 and 2011/12 might have been not substantial enough to produce meaningful changes in walking activity in older adults.

## Data Availability

Data from the Longitudinal Aging Study Amsterdam (LASA) are available for research. The LASA Steering Group has adopted a policy of sharing of data with interested researchers for specific research questions on aging-related issues. To obtain data, researchers need to submit an analysis proposal that is evaluated by the LASA steering group. Data are available for investigation under the condition that results of analyses will be made available to the research community through scientific reports or research papers, regardless of the results of the study. More information on data requests can be found on the LASA website: www.lasa-vu.nl. The geo-data that have been collected within the Geoscience and Health Cohort Consortium (GECCO) are available on request, provided that additional requirements of the original geo-data source holder are fulfilled and an agreement is made up with the GECCO steering group. More information on data requests can be found on the GECCO website: www.gecco.nl.

## Abbreviations

BV: Limited Liability Company
CI: Confidence Interval
dB (A): A-weighted decibels
e.g.: exempli gratia (for example)
GECCO: Geoscience and Health Cohort Consortium
GeoDMS: Geo Data and Model Software
IBM SPSS: International Business Machines Corporation Statistical Package for the Social Sciences
i.e.: id est. (in other words)
IQR: Interquartile range
LAPAQ: Longitudinal Aging Study Amsterdam Physical Activity Questionnaire
LASA: Longitudinal Aging Study Amsterdam
Lden: Level day-evening-night
LP: Limited Partnership
MMSE: Mini-Mental State Examination
n: number
NWA: Dutch Scientific Research Agenda
NWO: Netherlands Organisation for Scientific Research
PA: Physical activity
PBL: Planbureau voor de Leefomgeving (Netherlands Environmental Assessment Agency)
PM2.5: Particulate matter with a diameter of 2.5 μm
SD(s): Standard deviation(s)
USA: United States of America
ZonMw: The Netherlands Organisation for Health Research and Development
